# Albumin-Phthalocyanine Nanoconjugates as Platforms for Enhanced Photodynamic Cancer Therapy

**DOI:** 10.3390/ijms262311559

**Published:** 2025-11-28

**Authors:** Valentina I. Gorbacheva, Anastasiia O. Syrocheva, Ekaterina P. Kolesova

**Affiliations:** Research Center for Translational Medicine, Sirius University of Science and Technology, 354340 Sochi, Russia; tinafowl7@gmail.com (V.I.G.); syrocheva.ao@talantiuspeh.ru (A.O.S.)

**Keywords:** photodynamic therapy, photosensitizers drug delivery, nanocarriers, albumin nanoparticles, oxidative stress, lysosomal accumulation, necrotic cell death

## Abstract

This study investigates the enhancement of photodynamic therapy (PDT) efficacy through the encapsulation of platinum phthalocyanine (Pc) in albumin nanoparticles (ANP). Encapsulation of Pc in ANP) significantly enhances its biological effects in photodynamic therapy by increasing cellular uptake through receptor-mediated endocytosis and promoting lysosomal accumulation. This leads to marked lysosomal stress and regulated necrotic cell death pathway, while free Pc causes moderate oxidative stress with reversible apoptosis and autophagy. The enhanced phototoxicity of encapsulated Pc was evident across multiple cancer cell lines, especially aggressive phenotypes, whereas resistant lines showed lower sensitivity likely due to efficient ROS scavenging. Despite improved initial uptake, rapid lysosomal release and extracellular extrusion of Pc limit long-term intracellular retention. Morphological and gene expression analyses confirmed distinct cell death mechanisms between free and encapsulated Pc, underscoring the critical role of nanocarrier-mediated delivery in modulating oxidative stress and cellular response. These findings highlight the importance of nanoparticle design in optimizing PDT efficacy by effectively triggering necrotic cell death pathway.

## 1. Introduction

Photodynamic therapy (PDT) is a minimally invasive, clinically approved treatment modality for various cancers and precancerous conditions [1,2]. It relies on photosensitizers (PS) that are activated by specific wavelengths of light to generate reactive oxygen species (ROS), which induce oxidative stress and cause targeted cell death in diseased tissues [3]. The advantages of PDT include high selectivity for pathological cells, reduced systemic toxicity, and the ability to stimulate the immune system against tumors [4,5]. The efficacy of PDT largely depends on the photophysical and chemical properties of the photosensitizers used, prompting continuous efforts to discover and optimize new PS compounds [6,7,8,9]. Phthalocyanines (Pc), with their strong near-infrared absorption and high singlet oxygen quantum yield, are among the most promising PS classes [10]. As lipophilic molecules, Pc naturally tend to accumulate in cellular membranes [11]. However, this advantageous property is counteracted by a major limitation: their pronounced tendency to form aggregates in aqueous physiological media, a phenomenon known as aggregation-caused quenching (ACQ), which completely quenches their photodynamic activity [12].

To address the issue of aggregation, numerous studies have employed nanocarriers for Pc delivery [13,14]. A predominant strategy in PDT drug design has been to employ delivery systems that direct PS to mitochondria, a popular target for many photosensitizers beyond Pc, to efficiently trigger apoptosis [15,16,17,18]. While effective, this approach can be associated with elevated dark toxicity [19]. Furthermore, emerging evidence suggests that intentional lysosomal targeting offers a valuable alternative [20]. Lysosomal photodamage can not only maintain high PDT efficacy but also reduce non-specific toxicity and activate complementary, potent cell death pathways [21]. The challenge remains that Pc, even when delivered to lysosomes, predominantly resides there in an aggregated, photoinactive state. To overcome this, albumin nanoparticle (ANP) can be used to control the intracellular fate of Pc. ANPs offer distinct advantages, including exceptional biocompatibility, biodegradability, and a well-established safety profile [22,23]. Their delivery is enhanced by the EPR effect [24] and active targeting through gp60 and SPARC receptors [25], which are overexpressed in many tumors. A platform based on ANPs would not only enhance cellular uptake but also protect the Pc from aggregation, ensuring its delivery to lysosomes in a photoactive, monomeric form [26,27]. This dual-targeting approach—combining robust lysosomal photodamage with membrane disruption—is anticipated to activate complementary cell death pathways, culminating in a dramatic enhancement of phototoxicity. These properties make ANP an attractive third-generation photosensitizer delivery platform, advancing PDT towards higher specificity, efficacy, and safety in cancer treatment. There is also a growing number of studies investigating nanoparticles as autonomous multimodal sensitizers for theranostics and for the modification of photosensitizers’ optical properties [28,29].

Furthermore, by promoting widespread oxidative stress and the release of lysosomal contents, we aim to shift the mode of cell death into a more immunogenic form, potentially igniting a robust antitumor immune response [30]. Lysosomal accumulation of photosensitizers due to nanocarriers promotes robust oxidative stress and triggers regulated necrotic cell death that enhances antitumor immunity [31,32]. These advantages create potential for shifting PDT from localized treatments toward systemic therapy by improving delivery to metastatic and heterogeneous tumor sites, ultimately broadening clinical applications [33,34]. Thus, integrating advanced nanocarrier design promotes control over intracellular signaling cascades, harnessing cell death mechanisms essential for maximizing PDT effectiveness and transitioning toward personalized, systemically administered photodynamic cancer therapies.

This study focuses on the encapsulation of phthalocyanines in albumin nanoparticles and investigates how it affects the physicochemical and photophysical properties of free versus encapsulated PS, the generation of active oxygen species, cellular internalization, and cytotoxicity in vitro. Encapsulation in ANP significantly stabilizes PS molecules and enhances their cellular uptake, thereby amplifying oxidative stress and phototoxic effects. These insights are crucial, as understanding the molecular interactions and cellular responses induced by nanoparticle-delivered photosensitizers forms the fundamental basis for developing next-generation PDT systems for cancer and bacterial infections. Our results confirm that lysosomal delivery of Pc, coupled with its subsequent redistribution and unlocks a potent, multi-mechanistic cell death program. By elucidating the mechanistic interplay between nanoparticle delivery and photodynamic oxidative stress, this work aims to provide a robust foundational framework for optimizing photosensitizer design and delivery strategies to improve clinical PDT efficacy.

## 2. Results

### 2.1. Optical Properties of Pc as a Function of pH

**Figure 1:**
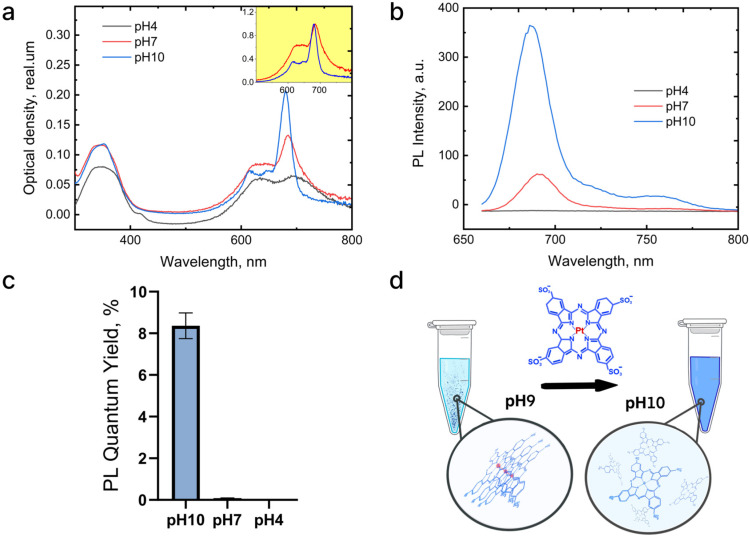
Optical properties of platinum sulfonated phthalocyanine (Pc) at different pH levels: (**a**) absorbance spectra with normalized spectra shown in the inset; (**b**) photoluminescence (PL) spectra with excitation wavelength of 640 nm; (**c**) PL quantum yield calculated relative to Rhodamine 6G; (**d**) schematic representation of Pc aggregation as pH decreases.

As shown in Figure 1, the absorbance and fluorescence spectra of platinum sulfonated phthalocyanine (Pc) align with the electronic structure typical of other phthalocyanines and exhibit strong dependence on environmental pH. The absorption spectra feature two characteristic bands at 640 nm and 350 nm (Figure 1a), with the shape of the 640 nm band demonstrating significant pH sensitivity (inset, Figure 1a). The fluorescence spectrum is characterized by a peak at 680 nm and a quantum yield of approximately 10% at alkaline pH (Figure 1b,c). When the pH decreases from 10 to physiological pH (~7), the fluorescence quantum yield drops sharply from 8% to 0.2% (Figure 1c). This pH-dependent variation in optical properties is common for molecules in this class and is typically associated with aggregation of the sensitizer molecules (Figure 1d).

### 2.2. Pc Loading into ANP

**Figure 2:**
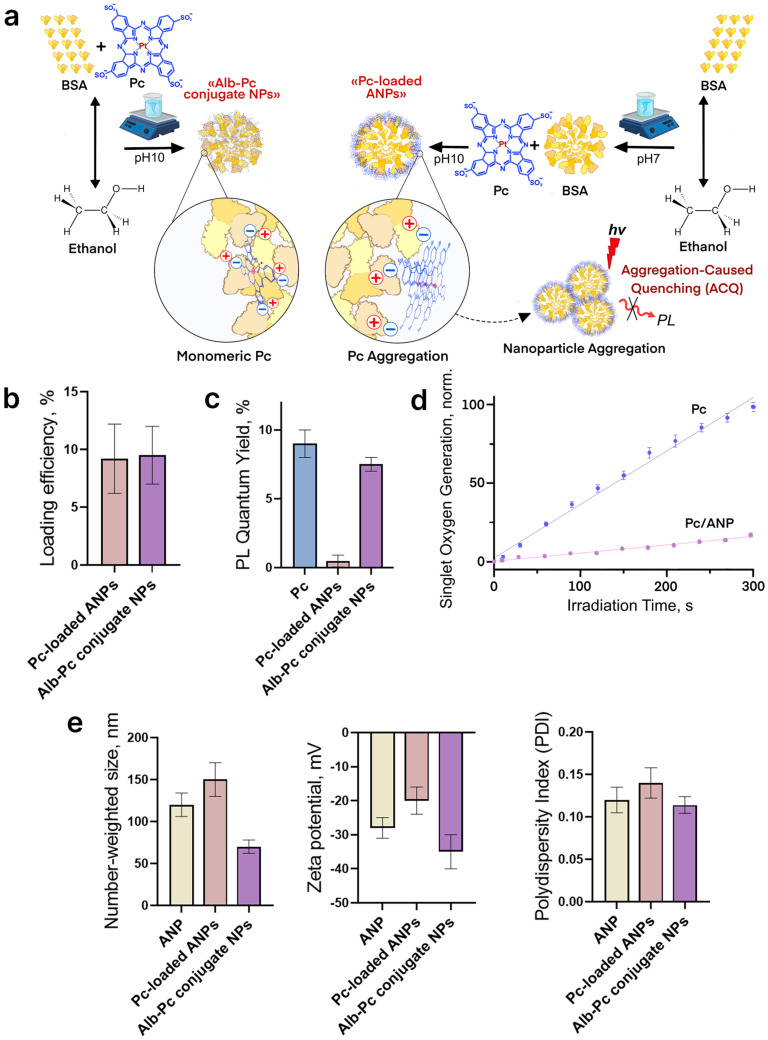
Loading of phthalocyanine (Pc) into albumin nanoparticles (ANPs): (**a**) Schematic representation of the loading methods used, including loading during nanoparticle synthesis (“Alb-Pc conjugate NPs”) and loading into preformed nanoparticles (“Pc-loaded ANPs”); (**b**) loading efficiency; (**c**) changes in Pc quantum yield upon loading; (**d**) singlet oxygen generation by free and encapsulated Pc, assessed using the chemical sensor Singlet Oxygen Sensor Green (SOSG); (**e**) physicochemical properties of the resulting Pc/ANP systems: hydrodynamic diameter (estimated by number), Zeta-potential and Polydispersity Index.

Two methods for Pc loading were evaluated: post-synthesis loading and loading during nanoparticle synthesis (Figure 2a). At neutral pH, the sulfonate groups of Pc acquire a negative charge, enabling electrostatic interactions with amino groups present on both free bovine serum albumin (BSA) and albumin nanoparticles. The loading efficiency was found to be independent of the loading method (Figure 2b). However, nanoparticles loaded during synthesis exhibited superior fluorescence properties, comparable to free Pc, suggesting reduced aggregation within these systems (Figure 2c and Figure S1 of Supplementary Information). Additionally, nanoparticles formed by loading Pc during synthesis («Alb-Pc conjugate NPs») demonstrated enhanced colloidal stability. In contrast, systems loaded post-synthesis showed aggregation four days after loading, which may be attributed to the influence of pH on ANPs physicochemical properties (Tables S1 and S2 of Supplementary Information).

As shown in Figure 2e, post-synthesis loading (“Pc-loaded ANPs”) caused a slight increase in particle size (from 123 to 151 nm) with a decrease in zeta potential (from −28 to −19 mV). In contrast, loading during synthesis resulted in smaller particle sizes (65 nm) than the original albumin nanoparticles, with an increased negative surface charge (−34 mV). Loading phthalocyanine during the synthesis process preserved the quantum yield of luminescence, indicating reduced aggregation and retention of photosensitizing properties. Combined with enhanced colloidal stability, these characteristics render such systems more promising for PDT. Therefore, this synthesis-stage loading method was employed in subsequent experiments.

Both free and encapsulated Pc demonstrated the capacity to generate singlet oxygen. Control experiments used concentrations of free Pc equivalent to those loaded in the nanocarriers. Reduced singlet oxygen generation efficiency is attributed primarily to suboptimal interactions between the sensor molecules and Pc (Figure 2d).

### 2.3. Pc/ANP Internalization

**Figure 3:**
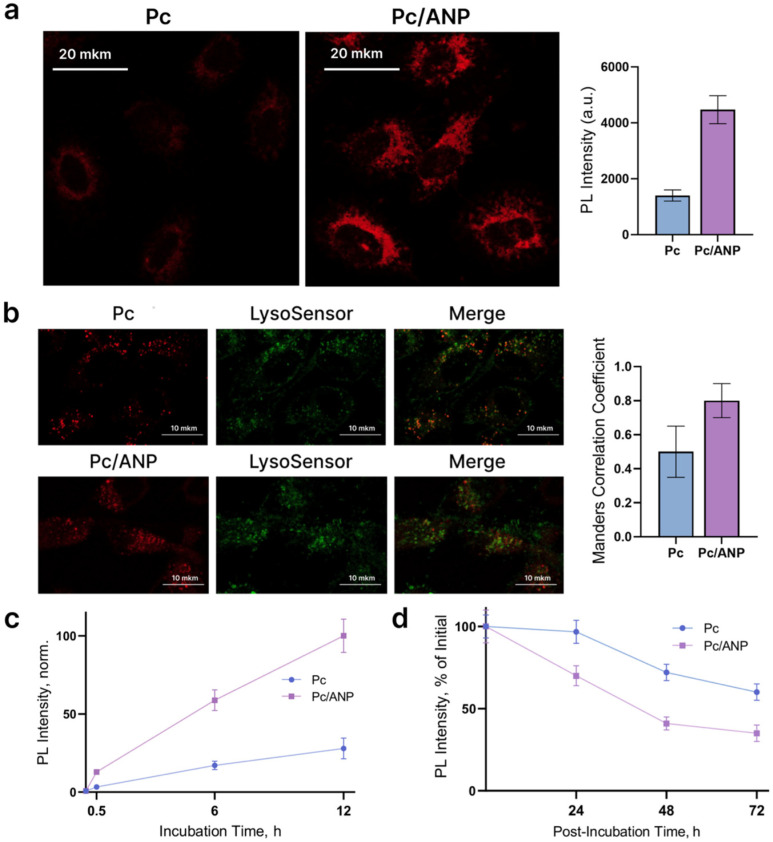
Internalization of Pc/ANP systems by SKBR-3 cells: (**a**) luminescence (PL) signal of Pc observed 12 h after cell treatment with free Pc and Pc/ANP systems. Scale bars indicate 20 µm; (**b**) localization of free and encapsulated Pc in lysosomes after 12 h of treatment (Pc—red, lysosomes—green, and colocalization—yellow in the merged channel. Scale bars indicate 10 µm. The histogram presents the corresponding Manders’ coefficient (tM1) values; (**c**) internalization kinetics and (**d**) release kinetics of free and encapsulated Pc measured by flow cytometry, represented as mean fluorescence intensity over time. In all experiments, the Pc concentration was 0.01 ng per cell.

It has been demonstrated that Pc/ANP systems exhibit significantly higher uptake into SKBR-3 cancer cells compared to free phthalocyanine. The fluorescence intensity of phthalocyanine delivered via albumin nanocarriers was approximately 3-fold greater, a result corroborated by flow cytometry analysis used to evaluate the internalization kinetics (Figure 3a). The analysis involving a larger number of cells is provided in the Supplementary Information (Figure S2 of Supplementary Information). Both free and encapsulated Pc can be internalized via endocytosis. Albumin nanoparticles tend to accumulate in lysosomes, whereas Pc primarily localizes at the periphery of the cytoplasm [35]. Pc/ANP systems exhibited greater colocalization with lysosomes compared to free Pc, as indicated by higher Manders’ colocalization coefficients (Figure 3b). Both free and encapsulated phthalocyanine showed similar time-dependent internalization profiles at 0.5, 6, and 12 h, with delivery via nanoparticles increasing internalization efficiency roughly 5-fold at each time point (Figure 3c). The release kinetics from cells followed comparable trends for both delivery types, with differences observed only during the first 24 h: free phthalocyanine showed no significant release, whereas Pc/ANP systems exhibited nearly a 30% decrease in fluorescence intensity, indicating photosensitizer release. At all assessed time points, Pc/ANP systems demonstrated greater efficiency in releasing phthalocyanine from cancer cells (Figure 3d). Notably, due to the enhanced internalization of nanoparticle delivery systems, intracellular photosensitizer concentrations remained higher at 72 h compared to free phthalocyanine (Figure S3 of Supplementary Information). Subsequent experiments employed a 12 h treatment with the delivery systems, followed immediately by irradiation for optimal therapeutic effect.

### 2.4. Pc/ANP Photodynamic Effect

**Figure 4:**
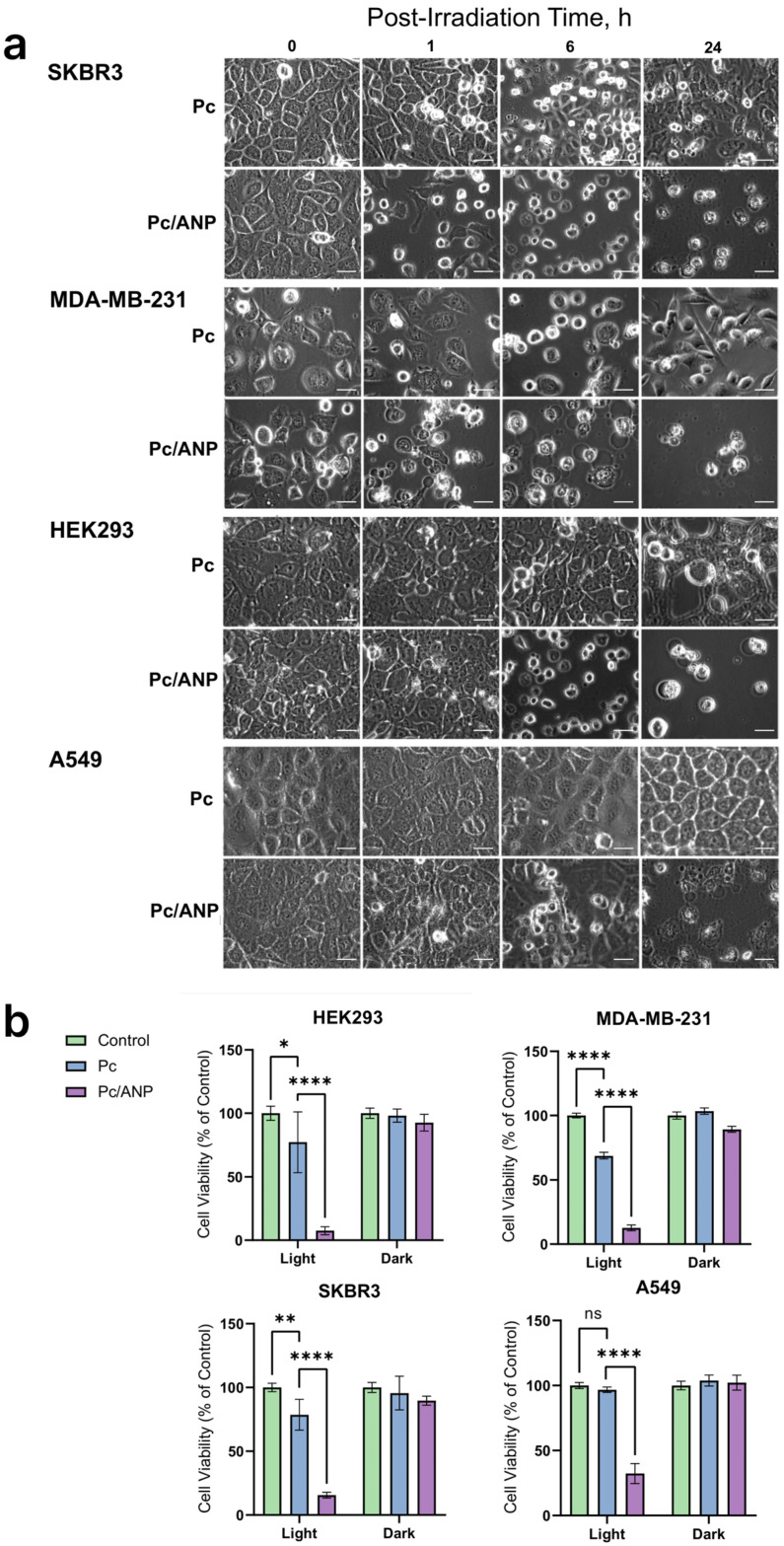
Effects of Pc/albumin nanoparticle (ANP) systems on A549, SKBR-3, HEK293, and MDA-MB-231 cells: (**a**) Morphological changes in cell cultures treated with free and encapsulated Pc, observed without irradiation and at 1, 6, and 24 h post-irradiation. Scale bars represent 20 µm (**b**) cell viability assessed 24 h after irradiation of cells treated with free and encapsulated Pc. Fluence of irradiation was 0.7 J/cm^2^. Results are presented as mean ± standard deviation (SD). Statistical significance was determined using one-way ANOVA followed by Dunnett’s test, with, * = *p* < 0.1, ** = *p* < 0.01, and **** = *p* < 0.0001. The Pc concentration was maintained at 0.01 ng per cell in all experiments.

To investigate differences in cell death mechanisms, morphological changes were examined in A549, SKBR-3, HEK293, and MDA-MB-231 cell lines at 1, 6, and 24 h post-irradiation following a 12 h incubation with either free or albumin nanoparticle-encapsulated Pc (Figure 4a). The delivery system played a crucial role in determining the cellular responses triggered by photosensitizer activation. Cells treated with free Pc exhibited rounding, formation of small surface blebs, and numerous intracellular vacuoles at 6 h, with partial morphological recovery by 24 h, suggesting an adaptive cellular response. Conversely, encapsulated phthalocyanine induced early (1 h) formation of large membrane blebs and cytoplasmic condensation in MDA cells, a phenotype that spread to SKBR-3, A549, and HEK293 lines by 6 h, culminating in widespread cell lysis and debris vesicle release into the culture medium at 24 h. Photodynamic cytotoxicity assessed at 24 h post-irradiation (Figure 4b) revealed high biocompatibility of both delivery systems in the absence of light (less than 10% cytotoxicity). Under experimental conditions, free Pc induced modest cytotoxicity (≤30%) across all lines, while nanoparticle delivery systems enhanced therapeutic efficacy approximately threefold. The lowest photodynamic effect was observed in A549 cells, whereas HEK293 cells showed the highest sensitivity. The lack of toxicity of the albumin nanoparticles (ANPs), including under irradiation conditions, is demonstrated in Figure S4 of the Supplementary Information.

The changes in mitochondrial ROS levels resulting from the photodynamic effect induced by free and encapsulated Pc were also analyzed. Cellular internalization did not produce significant changes in the fluorescence intensity or localization of the mitochondrial membrane potential-sensitive dye. No substantial changes were observed at 30 min post-irradiation. At 24 h post-irradiation, free Pc caused a significant alteration in intracellular dye localization and a decrease in fluorescence intensity. In contrast, encapsulated Pc showed a slight (~10%) increase in fluorescence intensity compared to the control and 0 min time points. Intracellular singlet oxygen generation assessed by SOSG is presented in Figure S5 of the Supplementary Information.

### 2.5. Cell Death Pathways Induced by PDT

To elucidate the key molecular determinants of cell death and interpret the phenotypic changes observed following photodynamic therapy, gene expression analysis was performed on SKBR-3 cells. The expression levels of genes associated with major cell death pathways were examined: apoptosis (effector gene *CASP3*), necroptosis (regulator *RIPK3*), pyroptosis (effector *GSDMD*), and ferroptosis (inhibitor *GPX4*). Additionally, genes involved in stress responses were measured, including lysosomal stress marker *LAMP1*, endoplasmic reticulum stress marker BIP, and oxidative stress markers *SOD2* and *GPX4*. Immunogenic consequences were assessed through expression of damage-associated molecular pattern (DAMP) signaling genes *CALR* and *HMGB1*. Gene expression was quantified at 3, 6, 12, and 24 h post-irradiation in cells treated with either free or encapsulated Pc, with TBP serving as the reference gene. These time points were selected to capture temporal variations in gene induction kinetics in response to oxidative stress and the initiation of different cell death programs. This comprehensive analysis provides insight into the molecular pathways activated by different photodynamic therapy formulations, aiding in understanding the mechanistic differences underlying therapeutic efficacy. The analysis performed does not allow for the precise determination of the mechanism of cell death. However, it enables the evaluation of whether the delivery systems (ANPs in this case) have a fundamental impact on the induced cell death pathway.

The expression of the CASP3 gene in the phthalocyanine-loaded nanoparticle (Pc/ANP) group peaked at 3–6 h post-irradiation, significantly exceeding the levels in the free phthalocyanine (Pc) group at these time points. While expression decreased in both groups by 12 h with no significant difference, a significant re-elevation was observed in the Pc/ANP group at 24 h. The RIPK3 gene demonstrated sustained upregulation in the Pc/ANP group across all post-irradiation time points (3, 6, 12, and 24 h) compared to the Pc group, with the most pronounced difference at 6 h. Similarly, LAMP1 expression was significantly higher in the Pc/ANP group from 3 to 24 h. In contrast, a statistically significant increase in GSDMD expression for the Pc/ANP group compared to Pc was only detected at the late 24 h time point. The dynamics of GPX4 expression were more complex; it was higher in the Pc/ANP group at 6 h, became lower than in the Pc group by 12 h, and then exhibited a sharp and significant rise in the Pc/ANP group at 24 h. BIP expression was significantly elevated in the Pc/ANP group solely at 12 h, whereas the CALR gene showed a powerful and sustained activation in the Pc/ANP group, with peaks at 6 and 24 h. HMGB1 mRNA levels were significantly higher in the Pc/ANP group during the mid-time points (3, 6, and 12 h) but equilibrated by 24 h. In contrast to the other genes, SOD2 expression showed no statistically significant intergroup differences at any time point. The results of the analysis are presented in Table 1.

## 3. Discussion

The absorbance spectrum of platinum phthalocyanine (Pc) features characteristic Q-bands in the visible region, typically ranging from approximately 669 to 698 nm. Incorporation of platinum as the central metal induces a pronounced hypsochromic shift in the Q-band by about 30 nm relative to zinc phthalocyanines (Figure 1a) [36]. This shift arises from alterations in the electronic structure due to the heavy atom effect exerted by platinum [37,38]. The Q-band is crucial for PDT applications as it corresponds to wavelengths with improved tissue penetration. The B-band appears within the blue to near-ultraviolet range, generally between 300 and 400 nm. These absorption bands originate from electronic transitions within the extensive π-conjugated macrocyclic phthalocyanine ring system [39]. Platinum phthalocyanines exhibit enhanced photostability and altered fluorescence behavior. The heavy atom facilitates intersystem crossing, increasing the triplet state population and singlet oxygen generation [40]. This is manifested as low fluorescence intensity and quantum yield (Figure 1b,c). The Q-band shape notably depends on pH (inset, Figure 1a), as phthalocyanines tend to aggregate in aqueous solutions, strongly influenced by acidity [41]. At acidic pH, increased protonation promotes the formation of H-type aggregates (face-to-face stacking). Aggregation of platinum sulfo-phthalocyanine involves π-π interactions between phthalocyanine rings and Pt-Pt bridging bonds [42], resulting in more stable aggregates with structural rigidity (Figure 1d). This aggregation causes fluorescence quenching and reduced photodynamic activity due to diminished reactive oxygen species production. This pH-triggered aggregation and instability must be accounted for in the design of Pc/ANP systems for cancer PDT, necessitating strategies for stabilization or controlled release.

Loading photosensitizers into nanocarriers such as ANP effectively addresses a key limitation of PDT by enhancing the stability and solubility of photosensitizer molecules. The high loading efficiency of phthalocyanines in albumin is primarily due to the strong affinity between their hydrophobic, aromatic macrocycles and the hydrophobic pockets of albumin, facilitating effective encapsulation or conjugation [43]. This binding not only stabilizes the photosensitizer but also leverages albumin’s natural transport capabilities to improve preferential uptake in cancer cells and prolong circulation time, thus amplifying therapeutic efficacy. For instance, octaphenyl-substituted erbium phthalocyanine demonstrated a high binding constant (~5.5 × 10^6^ M^−1^) with bovine serum albumin [44], indicating a robust complex formation. Two main approaches for loading drugs into albumin nanoparticles are used: during synthesis, where the drug distributes throughout the particle, and post-synthesis, where it localizes on the surface (Figure 2a). Both methods, when applied to encapsulate Pc, resulted in comparable loading efficiencies (~10%). The influence of solution pH during the loading of Pc was also analyzed, revealing that a decrease in pH leads to a reduction in loading efficiency (SI). It was found that the experimental design for assessing loading capacity has a greater impact than the aggregation state of Pc and interaction with albumin. Loading phthalocyanine during the synthesis of albumin nanoparticles initially appears less favorable because the singlet oxygen generated by Pc molecules embedded deep within the nanoparticles may have limited access to cellular components for efficient oxidation. Nevertheless, particles loaded during synthesis exhibited greater stability against aggregation and improved photoluminescence properties. Although an increase in fluorescence quantum yield was observed, it was not statistically significant (SI). Therefore, in our case, the formation of Pc-BSA conjugates did not alter the photosensitizer’s fluorescence properties. Conversely, encapsulating Pc molecules into preformed nanoparticles resulted in near-complete fluorescence quenching, likely due to Pc aggregation on the ANP surface mediated by stacking interactions during incubation. Drug loading commonly increases particle size, which was observed for Pc encapsulation into preformed ANPs; however, loading during synthesis at alkaline pH promotes a high negative surface charge, reducing final particle size. While standard ANP synthesis at alkaline pH followed by transfer to neutral pH causes gradual aggregation due to decreased surface charge modulus, synthesis in the presence of Pc appears to stabilize particles, enabling long-term storage at neutral pH. The complexation of Pc with ANPs not only prevents aggregation-induced quenching of the photosensitizer but also can enhance ROS generation upon irradiation. A comparable example is the encapsulation of chlorin e6 into albumin nanoparticles, which resulted in more than a twofold increase in singlet oxygen generation efficiency [45]. However, in our experiment using a singlet oxygen sensor, we observed a more than tenfold reduction in singlet oxygen generation efficiency. This decrease is partly attributed to spatial hindrance that limits interaction between the sensor molecules and the photosensitizer within the albumin particles (Figure 2). Despite this, the results confirm that the fluorescence and catalytic properties of phthalocyanine are preserved after encapsulation within nanoparticles.

In addition to enhancing stability, ANP as delivery system can significantly alter the pharmacokinetics and biodistribution of photosensitizers, particularly by increasing cellular internalization [46,47]. This effect is primarily mediated through albumin’s natural interactions with cell surface receptors such as gp60 and SPARC, facilitating receptor-mediated endocytosis and improving uptake efficiency compared to free photosensitizers [25]. For example, chlorin e6-conjugated albumin nanoparticles exhibit enhanced cellular uptake and phototoxicity due to these mechanisms [45]. Confocal microscopy showed a threefold increase in intracellular Pc concentration after 12 h of treatment with Pc/ANP, assuming equal fluorescence quantum yields of free and encapsulated forms (Figure 3a). Flow cytometry revealed a nearly fivefold increase in internalization rate with faster extrusion for Pc/ANP (Figure 3c). While free Pc distributes diffusely and remains stable over 24 h due to cell division, albumin-encapsulated Pc accumulates in lysosomes, saturating these compartments (Figure 3b). Free Pc can also utilize the endocytosis mechanism for internalization, similar to albumin nanoparticles, and localize in lysosomes shortly thereafter. Localization analysis revealed a significant increase in Pc accumulation when delivered via ANPs, with the Manders coefficient increasing from 0.5 to 0.8, which was an anticipated effect. A high degree of colocalization between ANPs and lysosomes was demonstrated in our previous studies [48]. The literature reports indicate that immediately after internalization, Pc may localize at the periphery of the cytoplasm [35], subsequently spreading deeper within the cell and interacting with various cellular membranes. Some studies demonstrated lysosomal localization of Pc [49] but suggested that at this time point, a portion of Pc interacts with the lysosomal membrane before undergoing redistribution within the cell. Lysosomal degradation releases phthalocyanine, which is rapidly extruded, leading to decreased fluorescence within 24 h (Figure 3d). Later, elimination rates of free and encapsulated photosensitizers converge due to dominant clearance mechanisms. Several studies support this rapid extrusion phenomenon. Our results confirm that although albumin nanocarriers enhance initial photosensitizer uptake, intracellular release and extrusion dynamics limit long-term retention. Although significant differences in cellular internalization were observed, further evaluation using in vivo mouse models is necessary to accurately assess the potential of albumin nanoparticles as targeted delivery systems. The increased internalization efficiency observed in vitro is likely due to variations in the cellular uptake mechanisms. Subsequent experiments involved 12 h treatment followed by irradiation to maximize intracellular photosensitizer concentration.

**Figure 5:**
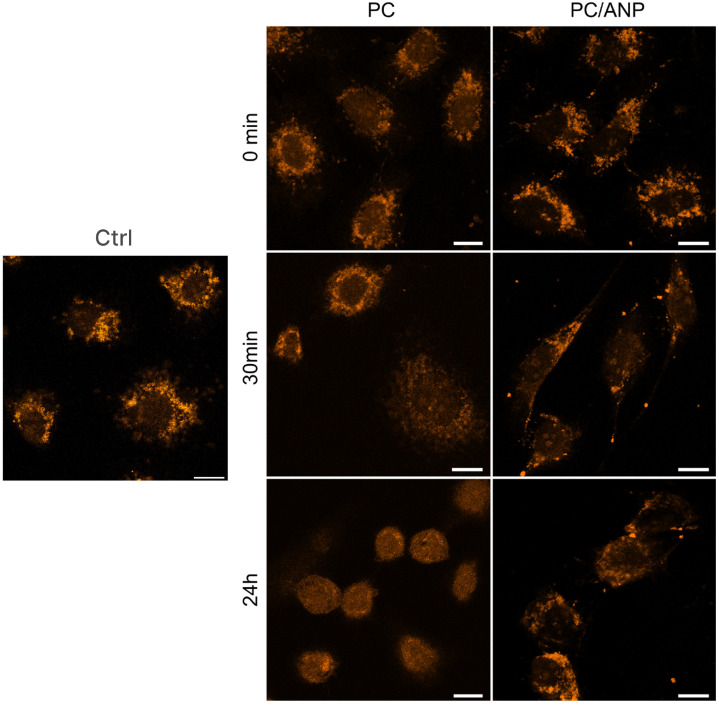
The effect of Pc/ANP systems on the mitochondrial reactive oxygen species (ROS) levels in SKBR-3 cells was assessed by confocal microscopy using LumiTracker Mito Orange CMTMRos at 30 min and 24 h post-irradiation. Fluence of irradiation was 0.7 J/cm^2^. Scale bars represent 10 µm.

Understanding the mechanisms of cell death induced by PDT is crucial, as apoptosis, necrosis, and autophagy pathways can all be triggered depending on photosensitizer localization and treatment conditions. Nanocarriers, such as albumin nanoparticles, can influence these death pathways by controlling photosensitizer delivery and subcellular localization, potentially enhancing therapeutic efficacy. Moreover, analyzing changes in cell morphology provides a valuable parameter for assessing PDT response and distinguishing between different cell death modalities. This integrated approach could improve evaluation and optimization of PDT protocols for transfer to clinical trials. Encapsulation of Pc within ANP rapidly induced the formation of large membrane blebs and cytoplasmic condensation, hallmark features of regulated necrosis, specifically necroptosis. In contrast, free Pc-treated cells exhibited apoptotic bodies and autophagosomes at 6 h post-irradiation, which dissipated by 24 h, indicating a moderate oxidative stress phenotype characterized by apoptosis and autophagy. The reversible nature of apoptosis and autophagy as a survival strategy in cancer cells aligns with observed partial morphological recovery at 24 h. The studied cell lines—SKBR-3, MDA-MB-231, A549, and HEK293—represent a range of cancerous and rapidly proliferating cells with diverse biological behaviors relevant to PDT. Encapsulation of Pc in albumin nanoparticles significantly increased cytotoxicity across all lines compared to free Pc, with the lowest toxicity observed in A549 cells, likely due to their efficient ROS scavenging [50] and diverse cell death signaling. Conversely, MDA-MB-231 cells showed the greatest toxicity difference between free and encapsulated Pc, reflecting their aggressive phenotype and enhanced susceptibility to nanocarrier-mediated delivery [51], which improves intracellular Pc accumulation and efficacy. The unexpectedly low viability of HEK293 cells, used here as immortalized non-cancerous cells with a high proliferation rate, may be attributed to two factors: the high efficiency of nanoparticle internalization and the cells’ heightened sensitivity to PDT. Several studies have demonstrated that albumin nanoparticle internalization in HEK293 cells occurs via a SPARC-dependent pathway, with uptake levels comparable to those observed in cancer cells (~75% as measured by confocal microscopy and flow cytometry) [52]. Numerous reports indicate that ANP-based platforms can affect both immortalized and cancerous cells, albeit with varying degrees of sensitivity. While these findings are encouraging, further in vivo studies are necessary to more definitively assess the targeting capabilities of ANP delivery systems. These findings underscore that cellular phenotype and nanocarrier delivery profoundly influence PDT outcomes. It is noteworthy that ANP-based systems demonstrated significantly lower singlet oxygen generation efficiency (Figure 2d) while exhibiting higher toxicity. This phenomenon may be attributed to both enhanced internalization of the photosensitizer upon ANP encapsulation reduced environmental quenching effects (pH level of cancer cell). Intracellular singlet oxygen generation was assessed using a sensor (Figure S5), revealing greater singlet oxygen production in cells treated with Pc/ANPs, which correlates with the observed toxicity of these systems. However, a pronounced increase in mitochondrial reactive oxygen species (ROS) levels was not detected (Figure 5). A slight increase in fluorescence intensity was observed following internalization of both free and encapsulated Pc, likely due to increased mitochondrial number or membrane potential. At 24 h post-irradiation, cells treated with free Pc displayed decreased localized Mito Orange CMTMRos staining, suggesting mitochondrial damage. In contrast, ANP-treated cells showed lysosomal accumulation of Pc without corresponding changes in mitochondrial membrane potential or mitochondrial ROS levels.

**Figure 6:**
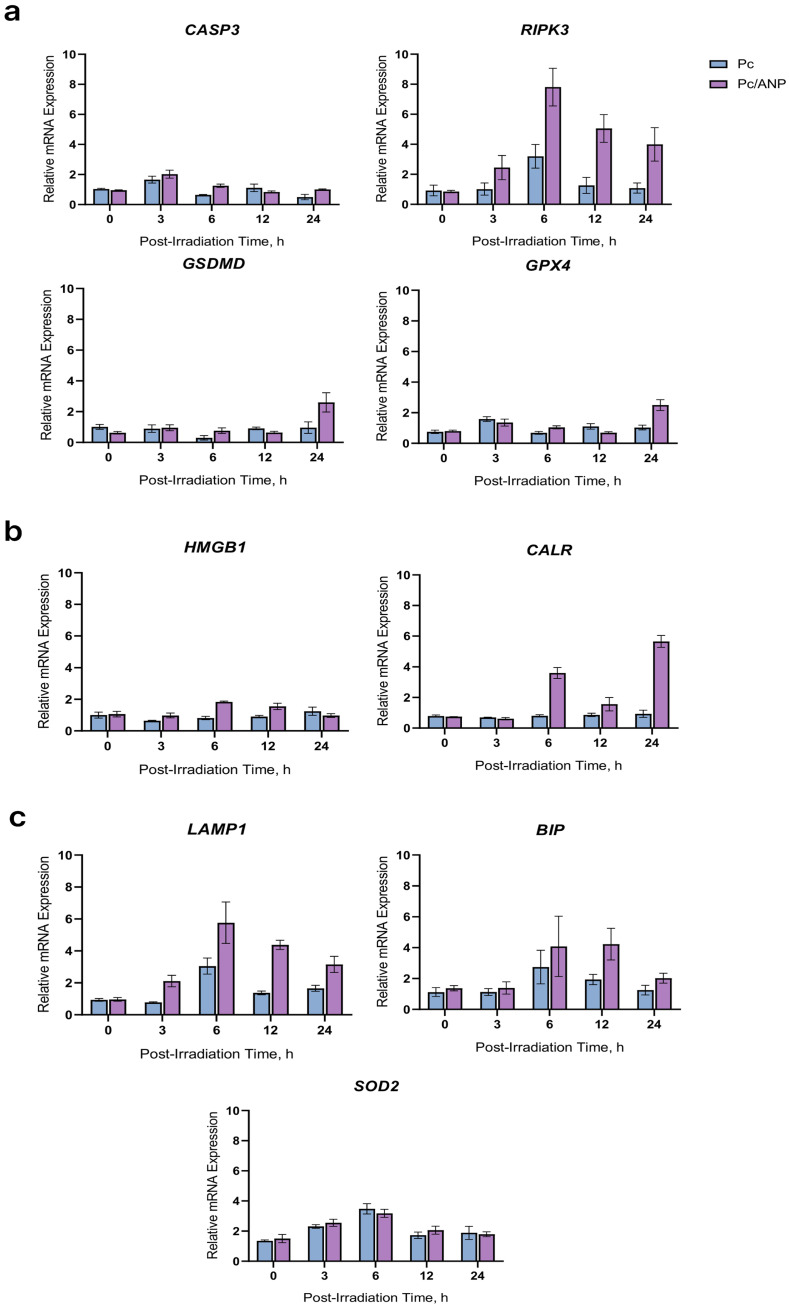
Changes in gene expression associated with (**a**) major cell death pathways, (**b**) immune response, and (**c**) stress response following photodynamic treatment with free phthalocyanine (Pc) and Pc encapsulated in albumin nanoparticles (Pc/ANP) were assessed after a 12 h treatment at a fluence of 0.7 J/cm^2^ irradiation. Measurements were taken without irradiation (0) and at 3, 6, 12, and 24 h post-irradiation. Each experiment included three biological and three technical replicates. Data are presented as mean ± standard deviation (SD).

**Figure 7:**
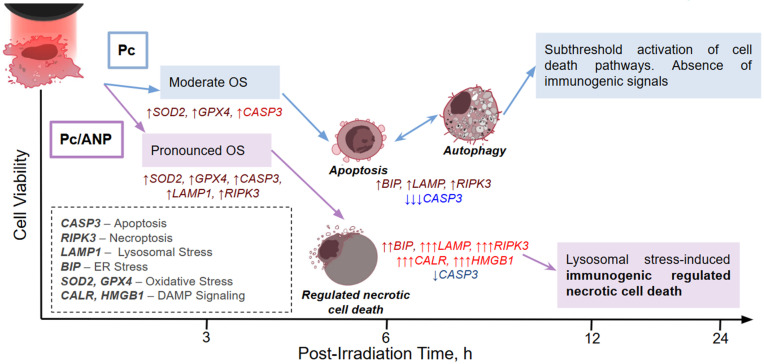
Schematic representation of the kinetics of various cell death markers induced by PDT with Pc. The dependence of the cell death mechanism on the encapsulation of the Pc within ANPs. Arrows indicate the dynamics (increase/decrease) and the magnitude of changes in gene expression.

Oxidative stress results from an imbalance between ROS production and cellular antioxidant defenses, causing damage to lipids, proteins, and nucleic acids. Traditional cell death forms in PDT—apoptosis, autophagy, and necrosis—are now joined by newly recognized regulated death pathways, such as mitotic catastrophe, paraptosis, pyroptosis, necroptosis, parthanatos, and ferroptosis [53]. These outcomes depend on photosensitizer localization, light dose, oxygen levels, and tumor genetics. Moderate oxidative stress induces apoptosis and autophagy, while higher stress triggers inflammatory necroptosis and pyroptosis, and extreme stress causes ferroptosis or parthanatos. Excessive oxidative stress leads to uncontrolled necrosis, promoting inflammation and fibrosis, which impairs immune response and treatment efficacy. Understanding these pathways is essential for optimizing PDT. The analysis of gene expression in SKBR3 cells at intervals of 3, 6, 12, and 24 h post-irradiation was conducted to capture the dynamic responses of genes involved in oxidative stress, given that different genes respond at varying speeds to such stress (Figure 6). Encapsulation of Pc in ANP (Pc/ANP) appears to trigger a cascade of interconnected cell death pathways initiated by intralysosomal stress, with necroptosis as the predominant death mechanism. The gene expression kinetics show early lysosomal destabilization (*LAMP1*), supporting the hypothesis of localized ROS generation within lysosomes. This is followed by sustained necroptosis activation (*RIPK3*) alongside progressing oxidative stress, evident from compensatory yet depleting induction of antioxidant genes (*SOD2*, *GPX4*). Concurrently, secondary endoplasmic reticulum stress (*BIP*) and late activation of membrane permeabilization effectors (*GSDMD*) occur. Terminal stages feature significant expression of immunogenic molecules (*CALR*, *HMGB1*), consistent with an immunogenic cell death phenotype. This sequence suggests that lysosomal damage may initiate a coordinated transition from organellar collapse to a regulated necrotic cell death associated with *RIPK3* upregulation, potentially enhanced by gasdermin-mediated membrane disruption, cumulatively promoting damage-associated molecular pattern (DAMP) release. A similar effect has been reported in photothermal therapy using gold nanoparticles, where necroptosis-like cell death via the RIPK1-RIPK3-MLKL pathway appears to amplify antitumor immune responses [54]. Comparable observations were made for a silicon (IV) phthalocyanine-based photosensitizer delivered by nanoparticles, which activated necroptosis-like pathways leading to DAMP release and enhanced antitumor immunity [55]. In contrast, free phthalocyanine induced only transient stress marker activation without evidence of an integrated response or immunogenic cell death; this was characterized by unstable *RIPK3* activation, suppression of the apoptotic cascade (reduced *CASP3*), and inhibited gasdermin-D signaling (*GSDMD*), without induction of key immunogenic mediators (*CALR*, *HMGB1*). Moderate regulation of antioxidant enzymes (*GPX4*, *SOD2*) suggested subcritical oxidative stress without depletion. Consistent with previous studies, free phthalocyanine primarily induced apoptosis via caspase-9 and caspase-3 pathways [56,57,58]. These data indicate a lack of threshold activation for effector death mechanisms, possibly due to diffuse cellular localization of the photosensitizer and lower uptake compared to encapsulated forms, resulting in an adaptive response without a clear pro-immunogenic phenotype (Figure 7). It should be noted that while these findings are indicative of necroptosis-like regulated necrosis and associated transcriptional changes, definitive evidence for immunogenic cell death (ICD) and antitumor immune activation requires further validation through direct ICD readouts (e.g., surface calreticulin exposure, ATP or HMGB1 release, dendritic cell activation, or in vivo vaccination studies).

## 4. Materials and Methods

### 4.1. Chemicals and Reagents

Three human cancer cell lines representing distinct malignancy and therapeutic resistance profiles were used in this study: SKBR-3, a HER2-enriched breast adenocarcinoma line characterized by aggressive growth and targeted therapy relevance; MDA-MB-231, a triple-negative breast cancer line notable for its invasiveness and resistance to conventional treatments; and A549, a human lung carcinoma line commonly employed to investigate respiratory cancer biology and drug response. HEK293T cells, derived from human embryonic kidney tissue, served as a non-cancerous control for comparative analyses. This diverse panel enables comprehensive evaluation of nanoparticle-mediated photodynamic therapy across tumor subtypes with varying sensitivities and molecular features. The cell lines derived from humans were purchased from the American Type Culture Collection. Cells were grown in the recommended medium (DMEM or RPMI) supplemented with 10% fetal bovine serum and 1% penicillin-streptomycin antibiotic mixture (all from Gibco, Waltham, MA, USA) at 5% CO2 and 37 °C in a humidified atmosphere containing 5% CO_2_. Cell lines were checked with the MycoReport (Evrogen, Moscow, Russia) and were free of contamination. Platinum phthalocyanine was obtained from the private collection of Vavilov’s Optical University (Saint-Petersburg, Russia). Bovine serum albumin and ethanol were purchased from Sigma-Aldrich (Saint Louis, MO, USA), while glutaraldehyde was purchased from PanReac AppliChem (Barcelona, Spain). All the reagents necessary to perform PCR analysis were obtained from Evrogen (Moscow, Russia). Singlet Oxygen Green Sensor, LumiTracker Lyso Green and LumiTracker^®^ Mito Orange CMTMRos were obtained from Lumiprobe (Moscow, Russia).

### 4.2. Synthesis and Characterization of Pc/ANP Systems

#### 4.2.1. ANP Synthesis

The synthesis was conducted according to a previously published protocol [48]. Briefly, the procedure involved two main steps: desolvation using ethanol and crosslinking with a minimal amount of glutaraldehyde. Sterility of the system was ensured by the use of a large volume of alcohol during synthesis. Once stable nanoparticles were formed, they were transferred into water while simultaneously removing free precursors, particularly glutaraldehyde. The resulting systems were stored in distilled, autoclaved water at 4 °C to maintain stability and sterility

#### 4.2.2. Pc Loading

There are two main approaches for loading substances into nanoparticles: during synthesis and after synthesis completion. When forming a Pc complex with pre-formed nanoparticles, the accumulation occurs primarily on the nanoparticle surface and within the subsurface porous layer. In contrast, when complexation takes place during synthesis, the Pc is distributed both on the exterior and within the internal matrix of the nanoparticles. This distinction influences loading efficiency, distribution, and subsequent photodynamic activity.

##### Loading in Synthesized ANP («Pc-Loaded ANPs»)

After ANP synthesis, an aqueous solution of Pc was added to the nanoparticle suspension at varying pH values (1 mg/mL ANP and Pc). Loading was performed via co-incubation under constant stirring at room temperature for 24 h. Subsequently, unbound Pc was removed by centrifugation. The resulting Pc-loaded ANPs were stored in aqueous solution at 4 °C to maintain stability.

##### Loading on ANP Synthesis Stage («Alb-Pc Conjugates NPs»)

In the first step, a solution of bovine serum albumin (20 mg/mL) was mixed with a Pc solution (10 mg/mL). Conjugation of the molecules was carried out under constant stirring at room temperature for 1 h in an aqueous solution at pH 10. Subsequently, the standard albumin nanoparticle (ANP) synthesis protocol was performed. The only significant difference was the pH of the solution, which was adjusted to pH 10 instead of pH 7 used in the standard ANP synthesis protocol.

#### 4.2.3. Systems Characterization

Optical properties, specifically absorption and photoluminescence spectra, were recorded using a HITA2J2-0013 U-3900 spectrophotometer (Hitachi, Chiyoda, Japan) and a Cary Eclipse spectrofluorimeter (Agilent, Mulgrave, Australia). The PL quantum yield was determined relative to Rhodamine 6G.

The physicochemical properties of the systems were analyzed using a Zetasizer Ultra analyzer (Malvern Instruments, Malvern, UK). For the measurements, 1 mL of sample at a concentration of 0.01 mg/mL was prepared, resuspended, and placed in a cuvette. Scattering measurements were conducted at a detection angle of 173°, optimal for detecting particles larger than 100 nm while minimizing the effects of multiple scattering, dust, and larger particles. The average particle size was calculated using the Stokes–Einstein equation based on the Brownian motion velocity inferred from fluctuations in scattered light intensity. The average size was converted to particle number concentration using Mie theory. Zeta potential measurements were obtained utilizing the Smoluchowski model.

To determine singlet oxygen generation, the fluorescent sensor Singlet Oxygen Sensor Green (SOSG) was used. Upon reaction with singlet oxygen, SOSG forms a fluorescent product exhibiting maximum emission at 525 nm. A solution of SOSG at 5 µM concentration was added to the sample cuvette, which was then irradiated with LED light at a power density of 2 mW/cm^2^. Fluorescence intensity of the sensor was periodically recorded during irradiation with excitation at 504 nm, reflecting singlet oxygen generation by the system as a result of light absorption and excitation.

The loading of Pc was quantified by measuring the decrease in free Pc concentration in the solution. This was achieved by comparing the optical density at the absorption maximum (660 nm) between the initial Pc solution and the supernatant obtained after centrifugation of Pc/ANP systems. The amount of Pc loaded into the nanoparticles was calculated based on this difference and used to determine the drug loading efficiency, defined as the mass of loaded Pc divided by the mass of dried Pc/ANP systems. This method was applied to both nanoparticles loaded during synthesis (“in process”) and those loaded after nanoparticle formation (“after”).

### 4.3. Systems Interaction with Cells

For all experiments, cells of lines A549, SKBR-3, HEK293, and MDA-MB-231 were seeded into Petri dishes or well plates. After 24 h to allow cell attachment, the plates were rinsed with PBS. The cells were then treated for 12 h with either free Pc or Pc/ANP at an equivalent Pc concentration of 0.01 ng per cell. Following a 12 h incubation, cells were washed with PBS and irradiated with 630 nm wavelength light at a total fluence of 0.7 J/cm^2^ and a power density of 2 mW/cm^2^ for 6 min. This irradiance is sufficient for investigating PDT effects as it activates the photosensitizer without causing excessive thermal damage. Minimal power settings were chosen to observe subtle molecular changes. Moreover, some studies suggest that lower irradiation energies hold greater potential for clinical translation of PDT. Various aspects of the interaction between the systems and the cells were analyzed at multiple time points post-irradiation. The majority of experiments were conducted using SKBR-3 cells.

#### 4.3.1. Analysis of Pc/ANP Cellular Internalization

The internalization efficiency was assessed in SKBR-3 breast cancer cells using confocal microscopy. 30 × 10^4^ cells were seeded into 3 cm Petri dishes with thin-bottom wells. Images were captured 12 h post-incubation using a LSM 980 Airyscan (Carl Zeiss, Oberkochen, Germany) confocal microscope.

The kinetics of internalization and release of free versus encapsulated Pc were analyzed by flow cytometry CytoFlex (Beckman Coulter, Brea, CA, USA). Internalization was quantified by measuring the median fluorescence intensity at 0.5, 6, and 12 h post-treatment. To study the release dynamics of Pc, its fluorescence intensity within cells was measured at 24, 48, and 72 h following a 12 h incubation and subsequent PBS wash.

#### 4.3.2. Analysis of Photodynamical Effect

The cytotoxic effects of the systems on cells were evaluated using the MTT assay, which is a standard protocol for such studies. Multiple cell lines, including A549, SKBR-3, HEK293, and MDA-MB-231, were used. 30 × 10^3^ cells were seeded per well in a 96-well plate with black walls. Cytotoxicity was assessed 24 h after irradiation, during which a specialized mask was applied to prevent light exposure to adjacent wells. For each control and treatment concentration, six replicate wells were used. The tetrazolium salt MTT was prepared at 5 mg/mL in PBS and added to SKBR-3 cells at 100 µL per mL of DMEM without serum or phenol red, following the Mosmann protocol. After a 1 h incubation at 37 °C, plates were washed with PBS, and DMSO was added to dissolve the dark-blue formazan crystals. The plates were then allowed to equilibrate at room temperature for several minutes before absorbance was measured at 570 nm using a CLARIOstar^®^ Plus reader (BMG Labtech, Ortenberg, Germany).

For analysis of morphological changes in cells induced by the photodynamic effect, a similar sample preparation protocol was used. Cell morphology was assessed at 1, 6, and 24 h post-treatment using an Axio Vert. A1 inverted microscope (Carl Zeiss, Oberkochen, Germany). For the analysis of mitochondrial ROS levels, cells were processed according to a standard protocol and stained at 0, 0.5, and 24 h using LumiTracker Mito Orange CMTMRos (Lumiprobe, Moscow, Russia) following the manufacturer’s instructions.

#### 4.3.3. Analysis of Cell Death Mechanism

For gene expression analysis, 30 × 10^4^ SKBR-3 cells were seeded into 3 cm Petri dishes and treated with the respective systems following the standard protocol. Gene expression was measured at 3, 6, 12, and 24 h post-treatment using Real-Time Quantitative Polymerase Chain Reaction (RT-qPCR). Total RNA was extracted from the cells with the PureLink™ RNA Kit (Invitrogen, Waltham, MA, USA) according to the manufacturer’s protocol. Complementary DNA (cDNA) was synthesized from messenger RNA (mRNA) using the cDNA MMLV kit (Evrogen, Moscow, Russia). For reverse transcription, one microgram of total RNA with an OD260/OD280 ratio between 1.7 and 2.0, determined by NanoDrop One (ThermoFisher, Waltham, MA, USA), was used. Human gene expression was quantified by RT-qPCR using the synthesized cDNA as template with 5X qPCRmix-HS (Evrogen) and gene-specific primers listed in Table 2.

PCR reactions were conducted in triplicate under the following conditions: an initial denaturation at 95 °C for 30 s, followed by 40 cycles of 95 °C for 5 s, 60 °C for 15 s, and 72 °C for 10 s, using the StepOne Real-Time PCR System (Applied Biosystems, Waltham, MA, USA). Gene expression levels were quantified using the comparative delta-delta Ct (ddCT) method, employing the TBP gene as the internal reference.

### 4.4. Statistical Analysis

Statistical analysis was performed using GraphPad Prism 9.5 for Windows (GraphPad Software, San Diego, CA, USA). For real-time PCR, three biological replicates were conducted, each containing three technical replicates per sample. Western blot analysis was performed three times using distinct biological replicates. Confocal microscopy data were collected from three biological replicates, capturing images from multiple areas of each preparation. The data were analyzed using the ANOVA method. Results are presented as mean ± SD for normally distributed data. Statistical significance was assessed using One-Way ANOVA followed by Dunnett’s post hoc test, with *p*-values < 0.05 considered significant. Statistical significance is denoted as * *p* < 0.05, ** *p* < 0.01, and *** *p* < 0.001.

## 5. Conclusions

Encapsulation of phthalocyanine within albumin nanoparticles (ANPs) significantly enhances photosensitizer stability, solubility, and delivery efficiency. Albumin nanoparticles substantially improve cancer cell uptake of phthalocyanine, facilitating lysosomal accumulation and rapid intracellular release. This results in heightened photodynamic cytotoxicity across various cancer cell lines, with distinct cell death pathways activated depending on whether phthalocyanine is free or encapsulated. Encapsulation induces necroptosis-like regulated necrosis associated with lysosomal destabilization and markers of immunogenic cell death, while free phthalocyanine mainly triggers moderate oxidative stress responses through apoptosis and autophagy.

These results highlight the promise of platinum phthalocyanine-loaded albumin nanoparticles as promising systems for photodynamic therapy, underscoring the critical role of nanocarrier-mediated delivery in optimizing therapeutic outcomes and modulating cell death mechanisms. Future studies should prioritize in vivo experiments to validate targeted delivery and to investigate the activation of the necrotic cell death pathway, aiming to enhance anti-cancer efficacy through combined therapeutic and immune-stimulatory effects.

## Figures and Tables

**Table 1:** Key findings on cell death pathway activation.

Pathway	Key Gene(s)	Effect of Pc/ANP vs. Pc	Temporal Profile
Necroptosis	*RIPK3*	Strong Induction	Early and Sustained
Apoptosis	*CASP3*	Induction	Early (peaks at 6 h)
ER Stress/ICD	*CALR*, *BIP*, *HMGB1*	Induction	Mid-phase (6–12 h)
Pyroptosis	*GSDMD*	Induction (Delayed)	Late (24 h)
Ferroptosis	*GPX4*	Biphasic Response	Complex (Oscillating)
Lysosomal Stress	*LAMP1*	Induction	Sustained

**Table 2:** Oligonucleotides used as primers for quantitative PCR are designated as forward (F) and reverse (R) primers.

Gene	Oligonucleotide	
*CASP3*	F	GGTGCTATTGTGAGGCGGTT
	R	TGAGAATGGGGGAAGAGGCA
*RIPK3*	F	CTACGATGTGGCGGTCAAGAT
	R	GTCCCAGTTCACCTTCTCGATAAC
*TNF-α*	F	CGAGTGACAAGCCTGTAGCC
	R	GGACCTGGGAGTAGATGAGGT
*GSDMD*	F	AGGACAGGCAAAGATCGCAG
	R	CACCTCAGTCACCACGTACA
*CALR*	F	AACCCCGAGTATTCTCCCGA
	R	GCTCAGCGTATGCCTCATCGT
*GPX4*	F	TTCCCGTGTAACCAGTTCG
	R	GCCCTTGGGTTGGATCTTCA
*HMGB1*	F	CGACTCTGTGCCTCGCTG
	R	TCCTCCCGACAAGTTTGC
*LAMP1*	F	GTGTCACGAAGGCGTTTTCAG
	R	TGTTCTCGTCCAGCAGACAC
*SOD2*	F	GCACTAGCAGCATGTTGAGC
	R	TTGATGTGAGGTTCCAGGGC
*TBP*	F	TGTATCCACAGTGAATCTTGGTTG
	R	GGTTCGTGGCTCTCTTATCCTC
*BIP*	F	GGAACCATCCCGTGGCATAA
	R	TGGTAGGCACCACTGTGTTC

## Data Availability

The original contributions presented in this study are included in the article/Supplementary Materials. Further inquiries can be directed to the corresponding author.

## Supplementary Materials

The following supporting information can be downloaded at: https://www.mdpi.com/article/10.3390/ijms262311559/s1.
