# Supplementary material for: Albumin-Phthalocyanine Nanoconjugates as Platforms for Enhanced Photodynamic Cancer Therapy
Source: Int J Mol Sci. 2025 Nov 28;26(23):11559. doi: 10.3390/ijms262311559 (PMC12692369; doi:10.3390/ijms262311559)
Supplement: Supplementary file 1 [file ijms-26-11559-s001.zip › ijms-3976992-supplementary.pdf]

Supplementary Information

Table S1. Effect of synthesis pH level on the characteristics of albumin nanoparticles

BSA Concentration	Ethanol volume (x water)	Size, nm	Zeta-potential, mV	PI index	pH
20	2x	127,7	-22,7	0,05	7
20	4x	107,1	-30,355	0,088	7
20	2x	84,16	-37,58	0,12935	10
20	4x	61,46	-35,67	0,21	10

Table S2. Colloidal stability of systems

after		In process	
0 days	3 days	0 days	3 days
150	340	71	75
0.14	0.42	0.11	0.13

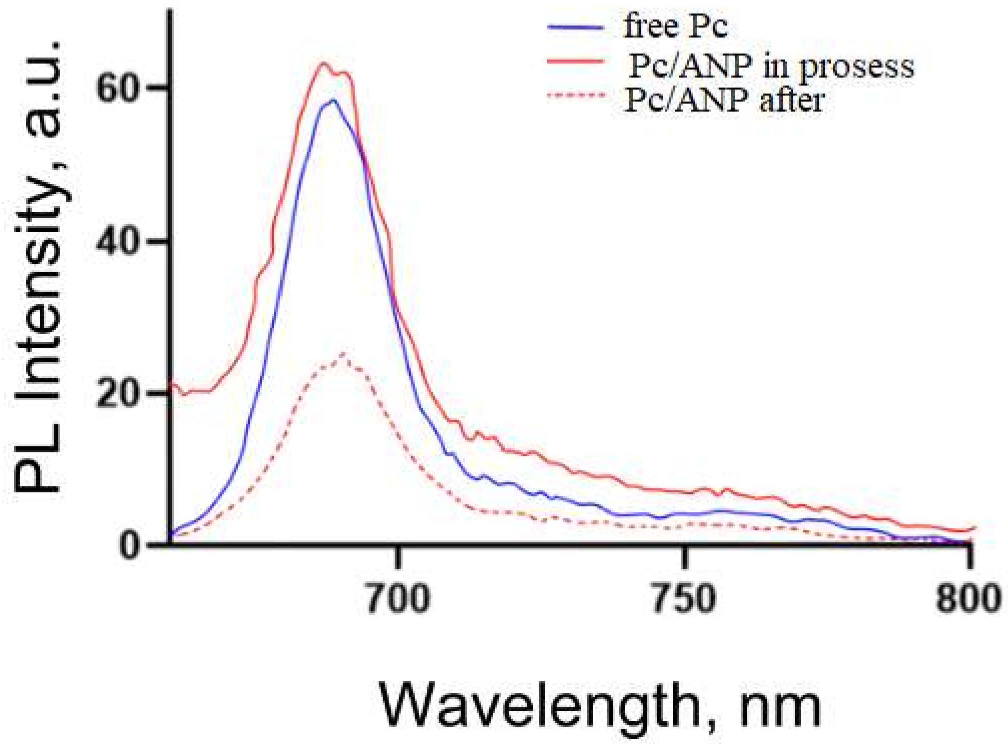

Figure S1. PL propertiees of free and encapsulated Pc

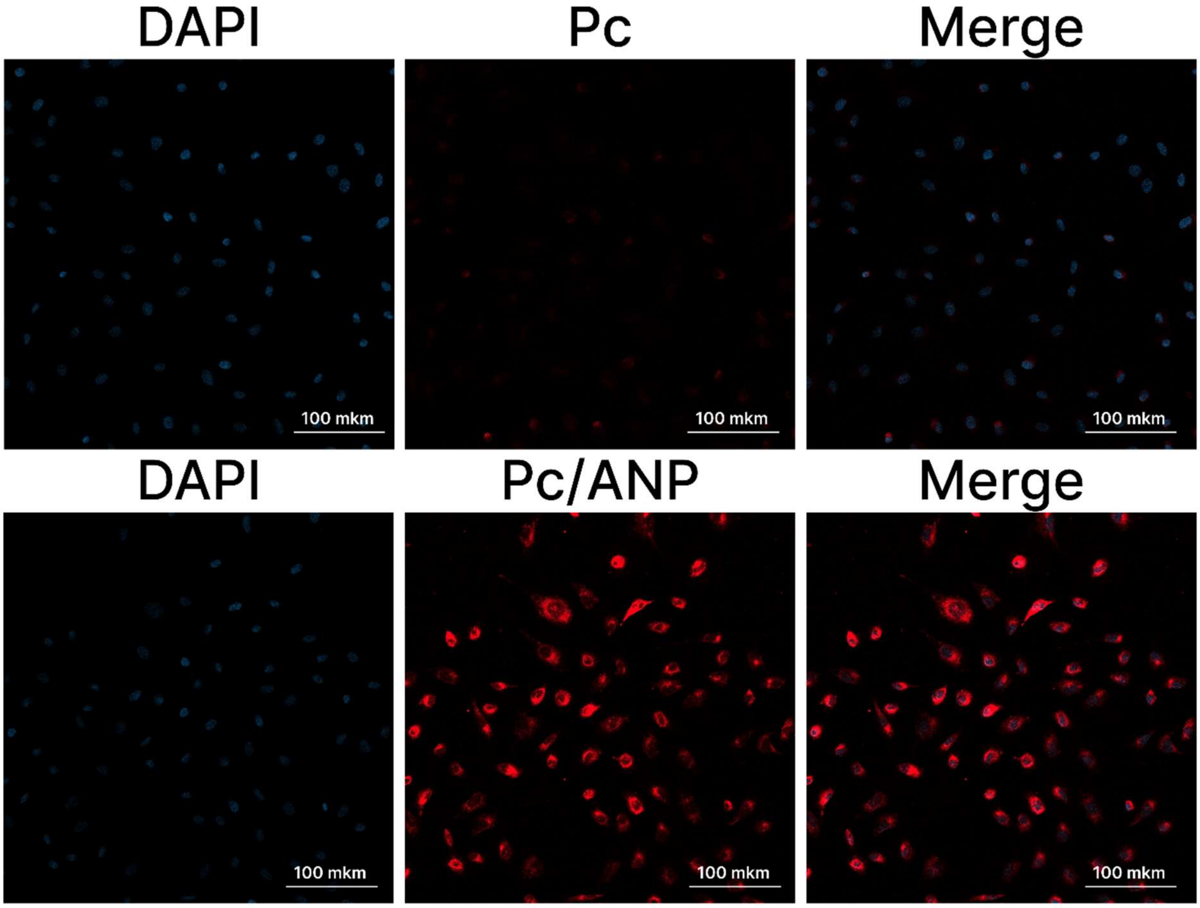

Figure S2. Photoluminescence (PL) images of SKBR-3 cells after 12 hours of treatment with Pc and Pc/ANP systems, acquired by confocal microscopy.

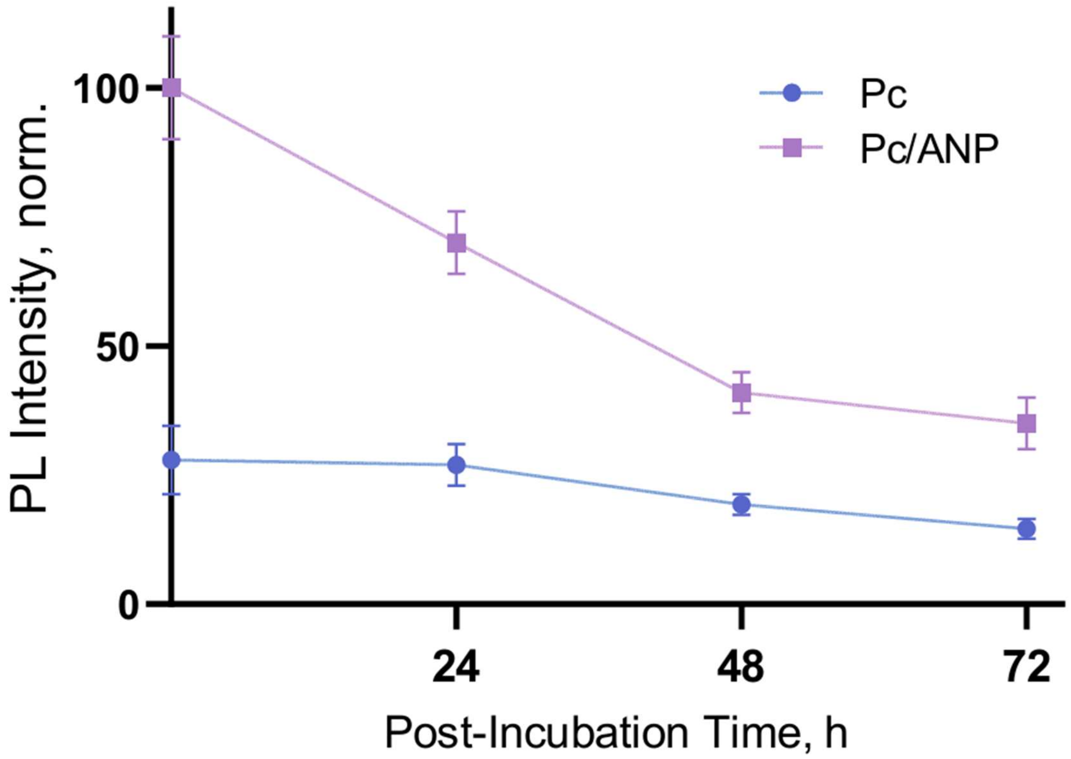

FigureS3. Release of Pc from SKBR-3 cells after 12 hours of treatment with Pc and Pc/ANP systems, acquired by FACS.

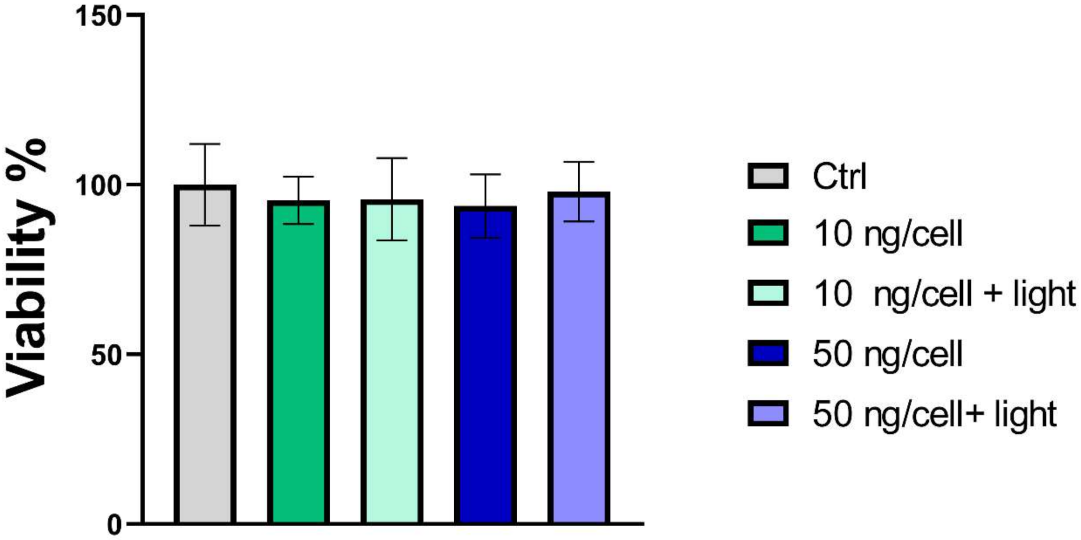

Figure S4. SKBR-3 cell viability assessed 48 hours after incubation with ANP and 24 h after irradiation. Results are presented as mean ± standard deviation (SD).

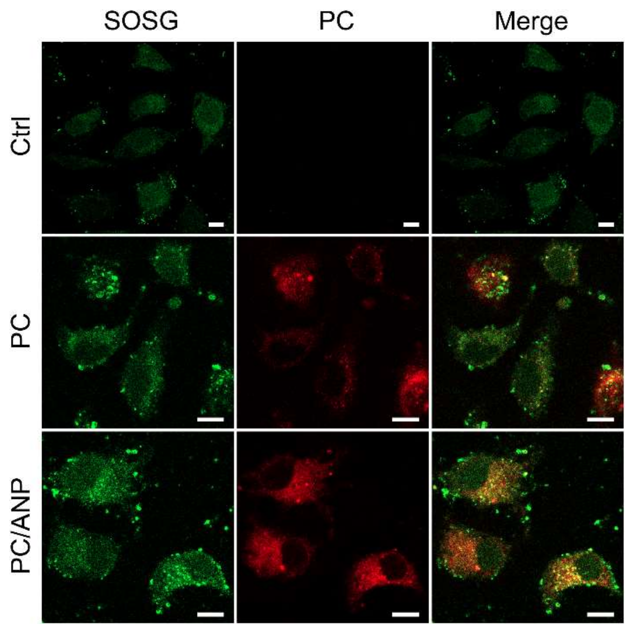

Figure S5. Intracellular singlet oxygen generation in SKBR-3 cells after 1 hour of irradiation was estimated using singlet oxygen sensor green (SOSG). The scale bar represents 10 μm.
